# Lack of associations of the opioid receptor mu 1 (OPRM1) A118G polymorphism (rs1799971) with alcohol dependence: review and meta-analysis of retrospective controlled studies

**DOI:** 10.1186/s12881-017-0478-4

**Published:** 2017-10-26

**Authors:** Xiangyi Kong, Hao Deng, Shun Gong, Theodore Alston, Yanguo Kong, Jingping Wang

**Affiliations:** 1 Department of Neurosurgery, Peking Union Medical College Hospital, Chinese Academy of Medical Sciences, No. 1 Shuaifuyuan Hutong, Dongcheng District, Beijing, 100730 People’s Republic of China; 2 Department of Anesthesia, Critical Care and Pain Medicine, Massachusetts General Hospital, Harvard Medical School, Harvard University, 55 Fruit Street, Boston, MA 02114-3117 USA; 3 Department of Breast Surgical Oncology, China National Cancer Center/Cancer Hospital, Chinese Academy of Medical Sciences and Peking Union Medical College, Chaoyangqu, Panjiayuan, Beijing, People’s Republic of China; 4 Department of Neurosurgery, Shanghai Institute of Neurosurgery, PLA Institute of Neurosurgery, Shanghai Changzheng Hospital, Second Military Medical University, 415 Fengyang Road, Shanghai, 200003 People’s Republic of China; 5 Department of Radiology, Brigham and Women’s Hospital, Harvard Medical School, 1249 Boylston St, Boston, MA 02215 USA

**Keywords:** Meta-analysis, Rs1799971, OPRM1 A118G, Polymorphism, Alcohol-dependence

## Abstract

**Background:**

Studies have sought associations of the opioid receptor mu 1 (OPRM1) A118G polymorphism (rs1799971) with alcohol-dependence, but findings are inconsistent. We summarize the information as to associations of rs1799971 (A > G) and the alcohol-dependence.

**Methods:**

Systematically, we reviewed related literatures using the Preferred Reporting Items for Systematic Reviews and Meta-Analyses (PRISMA) guideline. Embase, PubMed, Web of Knowledge, and Chinese National Knowledge Infrastructure (CNKI) databases were searched using select medical subject heading (MeSH) terms to identify all researches focusing on the present topic up to September 2016. Odds ratios (ORs) along with the 95% confidence interval (95% CI) were estimated in allele model, homozygote model, heterozygote model, dominant model and recessive model. Ethnicity-specific subgroup-analysis, sensitivity analysis, heterogeneity description, and publication-bias assessment were also analyzed.

**Results:**

There were 17 studies, including 9613 patients in the present meta-analysis. The ORs in the 5 genetic-models were 1.037 (95% CI: 0.890, 1.210; *p* = 0.64), 1.074 (95% CI: 0.831, 1.387; *p* = 0.586), 1.155 (95% CI: 0.935, 1.427; *p* = 0.181), 1.261 (95% CI: 1.008, 1.578; *p* = 0.042), 0.968 (95% CI: 0.758, 1.236; *p* = 0.793), respectively. An association is significant in the dominant model, but there is no statistical significance upon ethnicity-specific subgroup analysis.

**Conclusion:**

The rs1799971 (A > G) is not strongly associated with alcohol-dependence. However, there are study heterogeneities and limited sample sizes.

## Background

Alcohol-dependence is a common disorder involving psychological and physical alcohol-dependence despite frequent complications [1]. Based on DSM-IV criteria, no less than 3 out of 7 of the following criteria must be met during 12 months for alcohol-dependence: tolerance; use is continued in spite of knowledge of related harms; recreational, occupational or social pursuits are reduced or given up due to alcohol use; time is spent obtaining alcohol or recovering from effects; unsuccessful efforts or persistent desires to cut down on alcohol-use; use for longer periods or in larger amounts than intended; and withdrawal symptoms or clinically defined alcohol withdrawal syndrome [2]. There are around 76 million people suffered from alcohol dependence worldwide, which is one of the leading psychiatric disorders of adult patients [3]. Its etiology is still unclear [4]. There were some studies indicating heritability of this disorder (ranging from 49% to 64%) [5, 6]. Several studies concerning genome-wide or phenome-wide associations of alcohol dependence were listed in Table 1 [5, 7–11]. These researches suggested that genetic factors might influence the patient susceptibility to alcohol dependence.

**Table 1:** Previous studies about genome- or phenome-wide association studies of alcohol dependence

Association type	Author	Year	Country	PMID	Subjects number	Key findings
Genome-wide association studies	Gelernter J et al. [7]	2014	USA	24,166,409	16,087	1. They confirmed well-known risk loci mapped to alcohol-metabolizing enzyme genes, notably ADH1B in European-American (EA) and African-American (AA) populations and ADH1C in AAs, and identified novel risk loci mapping to the ADH gene cluster on chromosome 4 and extending centromerically beyond it to include GWS associations at LOC100507053 in AAs, PDLIM5 in EAs, and METAP in AAs. 2. They also identified a novel GWS association mapped to chromosome 2 at rs1437396, between MTIF2 and CCDC88A, across all of the EA and AA cohorts, with supportive gene expression evidence, and population-specific GWS for markers on chromosomes 5, 9 and 19.
	Xu K et al. [8]	2015	USA	26,036,284	9500	1. The results confirmed significant associations of the well-known functional loci at ADH1B with MaxDrinks in EAs and AAs. The region of significant association on chromosome 4 was extended to LOC100507053 in AAs but not EAs. 2. They also identified potentially novel significant common SNPs for MaxDrinks in EAs: rs1799876 at SERPINC1 on chromosome 1 and rs2309169 close to ANKRD36 on chromosome 2.
	Mbarek H et al. [5]	2015	Netherlands	26,365,420	7842	1. GWAS SNP effect concordance analysis was performed between GWAS and a recent alcohol dependence GWAS using DSM-IV diagnosis. The twin-based heritability of alcohol dependence-AUDIT was estimated at 60% (55–69%). 2. GCTA showed that common SNPs jointly capture 33% of this heritability. 3. The top hits were positioned within 4 regions (4q31.1, 2p16.1, 6q25.1, 7p14.1) with the strongest association detected for rs55768019.
	Polimanti R et al. [11]	2017	USA	26,458,734	5546	1. In the stage 1 sample, they observed 3 GWS SNP associations, rs200889048 and rs12490016 in EAs and rs1630623 in AAs and EAs meta-analyzed. 2. In the stage 2 sample, they replicated 278, 253 and 168 of the stage 1 suggestive loci in AAs, EAs, and AAs and EAs meta-analyzed, respectively. A meta-analysis of stage 1 and stage 2 samples identified 2 additional GWS signals: rs28562191 in EAs and rs56950471 in AAs
	Meyers JL et al. [9]	2017	USA	28,070,124	2382	1. Ten correlated SNPs located in an intergenic region on chromosome 3q26 were associated with fast beta (20–28 Hz) EEG power at *P* < 5 × 10–8. The most significantly associated SNP, rs11720469 is an expression quantitative trait locus for butyrylcholinesterase, expressed in thalamus tissue. 2. Four of the genome-wide SNPs were also associated with alcohol dependence, and two (rs13093097, rs7428372) were replicated in an independent AA sample. 3. Analyses in the AA adolescent/young adult subsample indicated association of rs11720469 with heavy episodic drinking (frequency of consuming 5+ drinks within 24 h).
Phenome-wide association studies	Polimanti R et al. [10]	2016	USA	27,187,070	26,394	1. They replicated prior associations with drinking behaviors and identified multiple novel phenome-wide significant and suggestive findings related to psychological traits, socioeconomic status, vascular/metabolic conditions, and reproductive health. 2. They applied Bayesian network learning algorithms to provide insight into the causative relationships of the novel ADH1B associations: ADH1B appears to affect phenotypic traits via both alcohol-mediated and alcohol-independent effects. They replicated the novel ADH1B associations related to socioeconomic status (household gross income and highest grade finished in school). 3. For CHRNA3-CHRNA5 risk alleles, they replicated association with smoking behaviors, lung cancer, and asthma. There were also novel suggestive CHRNA3-CHRNA5 findings with respect to high-cholesterol-medication use and distrustful attitude.

A relevant neurotransmitter system is related to endogenous opioids pathway [12]. Drinking alcohol can first increase levels of endogenous opioids (e.g. β-endorphin). Opioid reward system in return can elicit seeking additional alcohol. In addition, binding of μ-opioid receptors to β-endorphin could reinforce alcohol-dependence through increasing dopamine expressions at reward-centers [12] and then affect individual responses to alcohol. Therefore, genetic variations of OPRM1 might have an effect upon the risks of alcohol-dependence [13]. The rs1799971 is in the OPRM1 coding-area [13]. Though lots of researches have sought associations of the OPRM1 A118G- polymorphism with alcohol-dependence, there was no consensuses. [14] A Swedish group found that the A118G-polymorphism was connected to an 11% risk of alcohol dependence [15] while Bergen et al. found no significant association. [16] We were thus prompted to perform a meta-analysis to provide a full picture of current progress on this topic.

## Methods

### Article search and selection criteria

Two investigators searched CNKI, Embase, Web of Knowledge, and PubMed (up to Sep. 2016). Terms included “alcohol or alcoholic” and “rs1799971 or A118G or OPRM1”. Also, related references were scanned. Inclusion criteria and exclusion criteria are shown in Table 2.

**Table 2:** Inclusion criteria for this meta-analysis

Number	Inclusion criteria
1	Case-control studies.
2	The studies evaluated the associations between OPRM1 A118G polymorphism and alcohol dependence.
3	The studies included detailed genotyping data (total number of cases and controls, number of cases and controls with A/A, A/G, and G/G genotypes).
4	Studies focusing on human being.
Number	Exclusion criteria
1	The design of the experiments was not case-control.
2	The source of cases and controls, and other essential information were not provided.
3	The genotype distribution of the control population was not in accordance with the Hardy–Weinberg equilibrium (HWE).
4	Reviews and duplicated publications.

### Data extraction

We sought these information: authors’ names, publication-year, nation, ethnicity (Asian, Caucasian, or others), genotyping ways, *P* value for Hardy-Weinberg equilibrium (HWE),total numbers of controls and cases, controls and cases with OPRM1-A118G polymorphism, with A/A, A/G, and G/G genotypes, and control sources (population-based or hospital-based).

### Methodological qualities

Based on the methodological quality scale (see Table 3), 2 investigators estimated the study qualities independently. Disagreements were resolved by discussions. In the methodological quality assessment scale, five items (sample sizes, quality control of genotyping methods, source of controls, case representativeness, and HWEs) were checked. The scores range between 0 and 10, with 10 indicating highest quality.

**Table 3:** Scale for methodological quality assessment

Criteria	Score
1. Representativeness of cases	
RA diagnosed according to acknowledged criteria.	2
Mentioned the diagnosed criteria but not specifically described.	1
Not Mentioned.	0
2. Source of controls	
Population or community based	3
Hospital-based RA-free controls	2
Healthy volunteers without total description	1
RA-free controls with related diseases	0.5
Not described	0
3. Sample size	
> 300	2
200–300	1
< 200	0
4. Quality control of genotyping methods	
Repetition of partial/total tested samples with a different method	2
Repetition of partial/total tested samples with the same method	1
Not described	0
5. Hardy-Weinberg equilibrium (HWE)	
Hardy-Weinberg equilibrium in control subjects	1
Hardy-Weinberg disequilibrium in control subjects	0

### Statistical analysis

This analysis was in accord with the PRISMA checklist and guideline. ORs were computed in 3 steps: 1) for given individuals that have “B”, we computed the odds that the same individuals have “A”; 2) for given individuals that do not have “B”, we computed the odds that the same individuals have “A”; and 3) we divided the odds from step 1 by the odds from step 2, getting the ORs. The pooled ORs were estimated and used for comparisons in the 5 genetic models mentioned above. Ethnicity-specific subgroup-analyses were also made. To estimate the heterogeneities, we performed the I^2^ tests, Labbe plots, and Cochran’s Q-tests (see Table 4). As it seems likely that there are considerable phenotypic variations between populations in the different studies, we did all these analyses using the random-effects model. By contour-enhanced funnel plots and sensitivity-analysis plots (Table 4), we did publication-bias and sensitivity tests.

**Table 4:** The statistical methods used in this meta-analysis and there explanation

Statistic means	Goals and Usages	Explanation
Labbe plot	To evaluate heterogeneity between the included studies	In Labbe figure, if the points basically present as a linear distribution, it can be taken as an evidence of homogeneity.
Cochran’s Q test	To evaluate heterogeneity between the included studies	Cochran’s Q test is an extension to the McNemar test for related samples that provides a method for testing for differences between three or more matched sets of frequencies or proportions. Heterogeneity was also considered significant if *P* < 0.05 using the Cochran’s Q test.
I^2^ index test	To evaluate heterogeneity between the included studies	The I^2^ index measures the extent of true heterogeneity dividing the difference between the result of the Q test and its degrees of freedom (k – 1) by the Q value itself, and multiplied by 100. I^2^ values of 25%, 50% and 75% were used as evidence of low, moderate and high heterogeneity, respectively.
Sensitivity analysis	To examine the stability of the pooled results	A sensitivity analysis was performed using the one-at-a-time method, which involved omitting one study at a time and repeating the meta-analysis. If the omission of one study significantly changed the result, it implied that the result was sensitive to the studies included.
Contour-enhanced funnel plot	Publication bias test	Visual inspection of the Contour-enhanced funnel plots was used to assess potential publication bias. Asymmetry in the plots, which may be due to studies missing on the left-hand side of the plot that represents low statistical significance, suggested publication bias. If studies were missing in the high statistical significance areas (on the right-hand side of the plot), the funnel asymmetry was not considered to be due to publication bias

A value of *P* < 0.01 was deemed of statistical significance. Statistical-analyses were conducted with Review Manager 5.3 and STATA 13.0.

## Results

### Search results and study characteristics

**Fig. 1:**
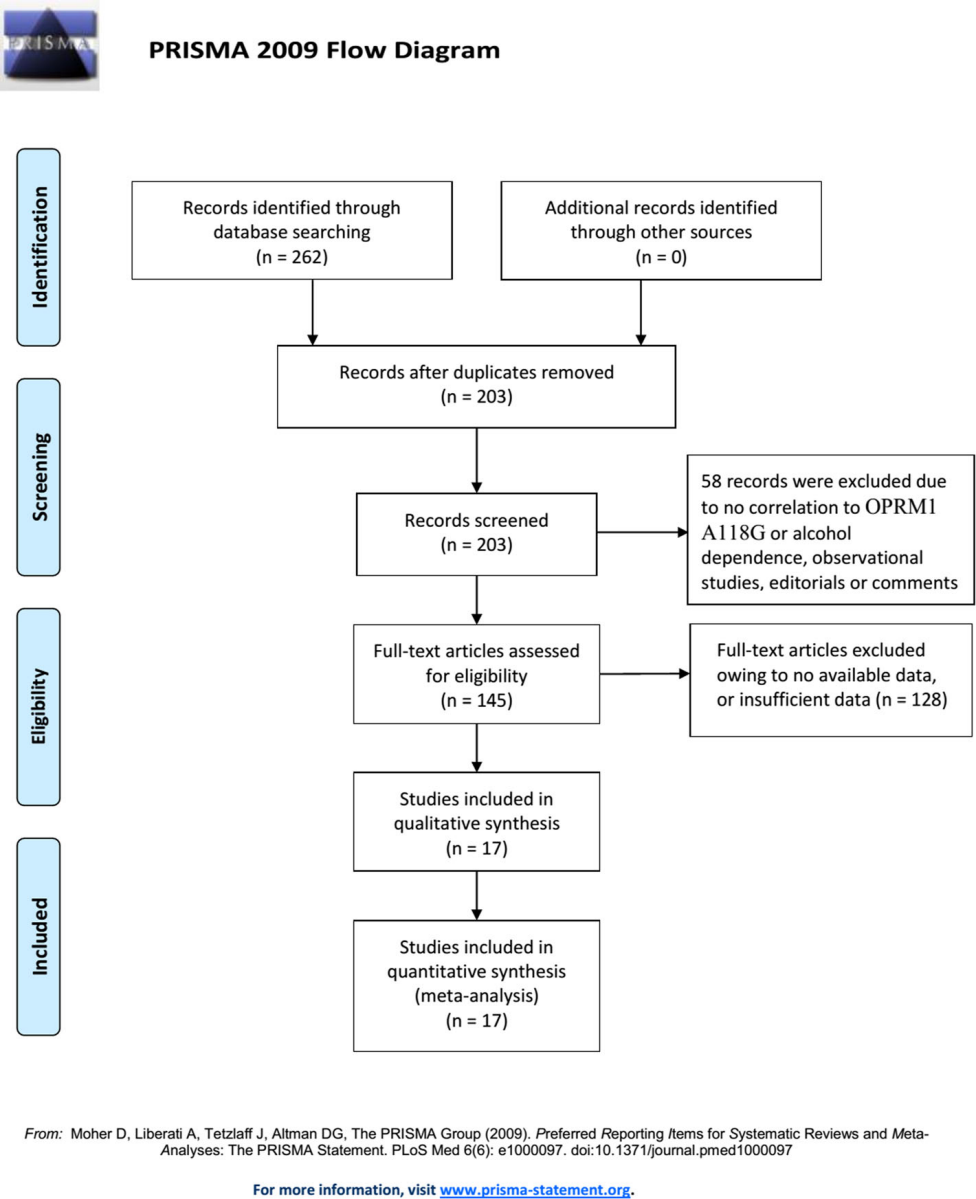
Literature search and selection of articles

Figure 1 shows the processes of the literature-searching. 17 studies with 9613 patients were included. [15–31] Nine studies involved Caucasian subjects and were done in the USA, [15, 16, 24, 28, 30] Germany, [19, 22, 27] and Spain [18] (8026 subjects in total). Eight involved Asian subjects and were done in China, [23, 26, 29, 31] India, [17] Japan, [25] and Korea [20, 21] (1587 subjects in total). Fourteen studies were written in English, [15–25, 27, 28, 30] and three were in Chinese. [26, 29, 31] Alcohol dependence was defined by drinking history. Genotyping methods used included direct sequencing, polymerase chain reaction-restricted fragment length polymorphisms (PCR-RFLP), Puregene™ kit or standard phenol-chloroform method, TaqMan assay, and fluorescence resonance energy transfer method. Ten matchings for the controls were population-based, [15, 16, 18, 24–29, 31] 3 were hospital-based, [20–22] and 4 were mixed. [17, 19, 23, 30] The characteristics and methodological qualities are in Table 5.

**Table 5:** Characteristics of studies included in the meta-analysis

Author	Year	Country	Ethnicity	Disease type	Genotyping	Source of controls	Alcohol-dependence (n)				Controls (n)				P for HWE	Quality
							Total	AA	AG	GG	Total	AA	AG	GG		
Bergen et al.	1997	USA	Caucasian	Alcohol-dependence	Direct sequencing and PCR-RFLP	Population-based	160	123	35	2	264	204	59	1	0.1285	7
Sander et al.	1998	German	Caucasian	Alcohol-dependence	PCR-RFLP	Population-based	327	261	62	4	340	289	49	2	0.9606	6
Franke et al.	2001	German	Caucasian	Alcohol-dependence	Direct sequencing and PCR-RFLP	Mixed	221	170	50	1	365	284	74	7	0.4024	8
Schinka et al.	2002	USA	Caucasian	Alcohol-dependence	Puregene™ kit or standard phenol-chloroform method	Population-based	179	152	27	0	297	220	73	4	0.4531	7
Kim et al.	2004	Korea	Asian	Alcohol-dependence	PCR-RFLP	Hospital-based	100	46	47	7	128	54	53	21	0.2014	8
Kim et al.	2004	Korea	Asian	Alcohol-dependence	PCR-RFLP	Hospital-based	112	37	61	14	140	68	57	15	0.5582	7
Loh et al.	2004	China Taiwan	Asian	Alcohol-dependence	PCR-RFLP	Mixed	154	59	77	18	146	70	56	20	0.1136	8
Bart et al.	2005	USA	Caucasian	Alcohol-dependence	PCR-RFLP	Population-based	389	299	90		170	147	23		Not available	8
Nishizawa et al.	2006	Japan	Asian	Alcohol-dependence	PCR-RFLP	Population-based	64	12	37	15	74	26	33	15	0.4493	8
Zhang et al.	2006	USA and Russia	Caucasian	Alcohol-dependence	PCR-RFLP	Mixed	318	246	68	4	338	256	78	4	0.4713	7
Deb et al.	2010	India	Asian	Alcohol-dependence	PCR-RFLP	Mixed	53	16	32	5	82	44	30	8	0.3967	8
Miranda et al.	2010	USA	Caucasian	Alcohol-dependence	TaqMan assays	Population-based	27	13	14		160	134	26		> 0.05	8
Dou et al.	2011	China	Asian	Alcohol-dependence	PCR-RFLP	Population-based	118	48	53	17	218	74	110	34	0.5127	6
Koller et al.	2012	Germany	Caucasian	Alcohol-dependence	Fluorescence resonance energy transfer method	Hospital-based	1845	1461	353	31	1863	1417	419	27	0.5275	9
Huang et al.	2012	China	Asian	Alcohol-dependence	PCR-RFLP	Population-based	45	33	11	1	45	33	12	0	0.3021	6
Francesc	2015	Spain	Caucasian	Alcohol-dependence	PCR-RFLP	Population-based	630	425	190	15	133	101	30	2	0.893	7
Jin	2015	China	Asian	Alcohol-dependence	PCR-RFLP	Population-based	58	41	12	5	50	39	9	2	0.1487	7

### Meta-analysis results

**Fig. 2:**
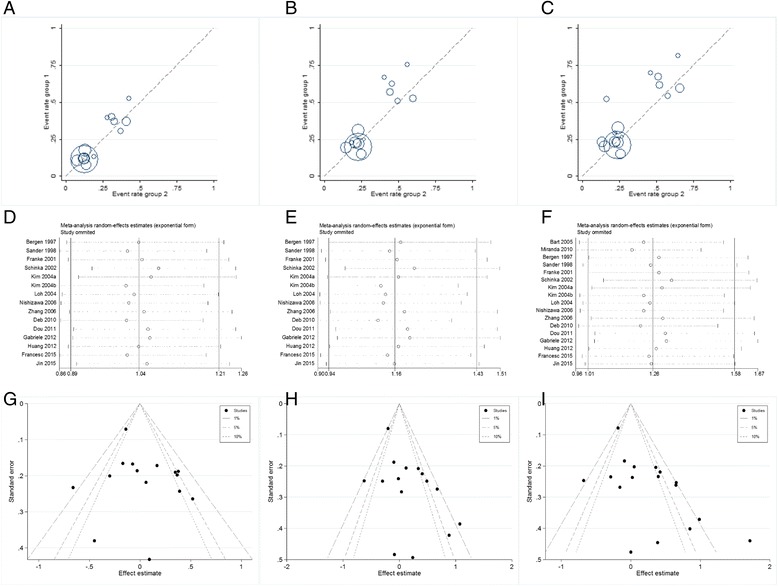
Labbe plots, sensitivity analysis plots, and contour-enhanced funnel plots of the included studies focusing on the association of the OPRM1 A118G polymorphism with alcohol dependence risk. Labbe plots in allele model (**a**), heterozygote model (**b**), and dominant model (**c**). Sensitivity analysis in allele model (**d**), heterozygote model (**e**), and dominant model (**f**). Contour-enhanced funnel plots in allele model (**g**), heterozygote model (**h**), and dominant model (**i**)

**Fig. 3:**
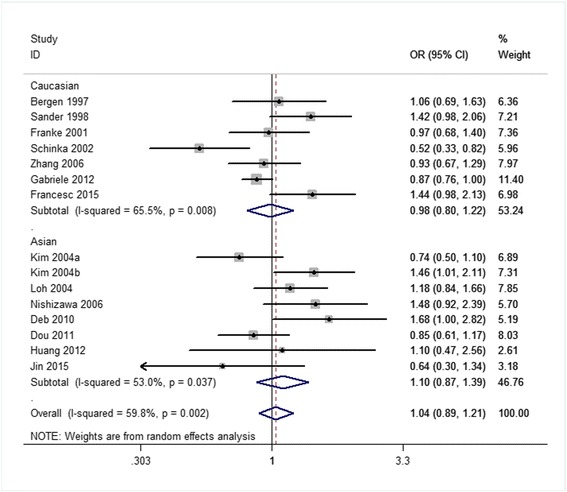
Forest plots (individual and pooled effects with 95% CI) regarding the association of the OPRM1 A118G polymorphism with alcohol dependence in the allele model

**Fig. 4:**
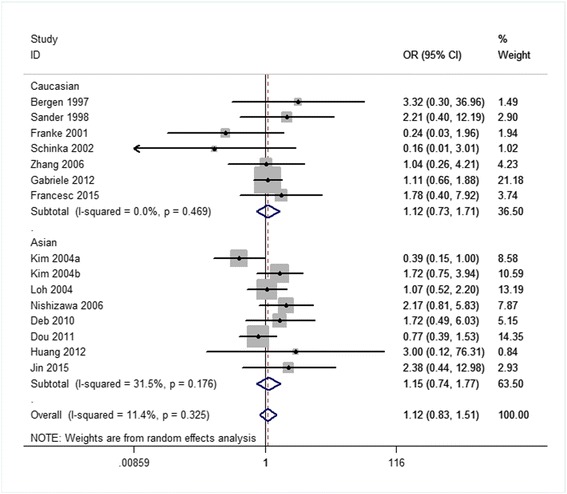
Forest plots (individual and pooled effects with 95% CI) regarding the association of the OPRM1 A118G polymorphism with alcohol dependence in the homozygote model

**Fig. 5:**
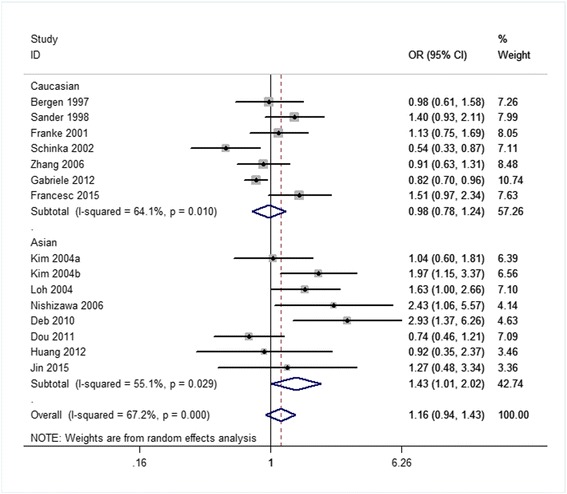
Forest plots (individual and pooled effects with 95% CI) regarding the association of the OPRM1 A118G polymorphism with alcohol dependence in the heterozygote model

**Fig. 6:**
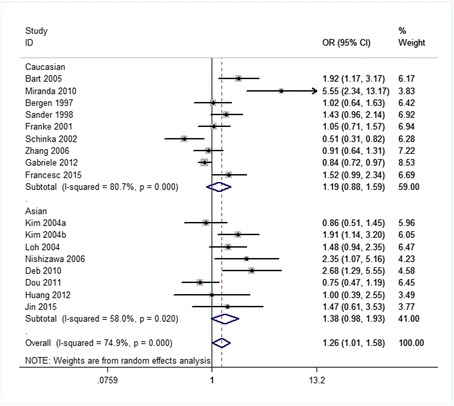
Forest plots (individual and pooled effects with 95% CI) regarding the association of the OPRM1 A118G polymorphism with alcohol dependence in the dominant model

**Fig. 7:**
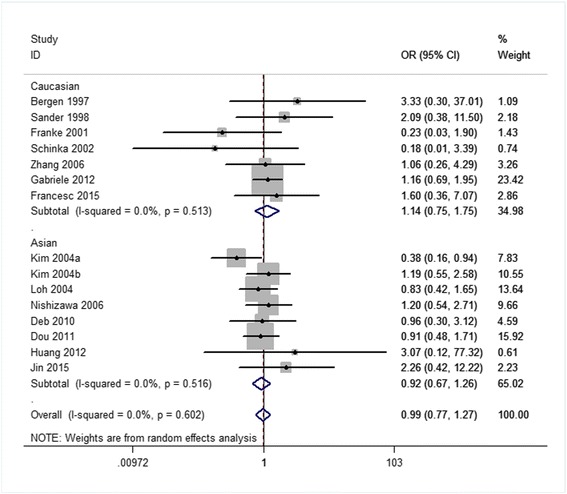
Forest plots (individual and pooled effects with 95% CI) regarding the association of the OPRM1 A118G polymorphism with alcohol dependence in the recessive model

Related results are listed in Table 6. The Labbe plots are as Fig. 2a–c. Overall, statistically significant associations of OPRM1-A118G polymorphism with alcohol-dependence was detected only in the dominant model (OR 1.261, 95% CI 1.008, 1.578; *p* = 0.042; Fig. 6). In the other four models, any associations were not significant (allele model: OR 1.037, 95% CI 0.890, 1.210; *p* = 0.640; Fig. 3; homozygote model: OR 1.074, 95% CI 0.831, 1.387; *p* = 0.586; Fig. 4; heterozygote model: OR 1.155, 95% CI 0.935, 1.427; *p* = 0.181; Fig. 5; recessive model: OR 0.968, 95% CI 0.758, 1.236; *p* = 0.793; Fig. 7).

**Table 6:** The results of meta-analysis for various genotype models

Genetic model			Heterogeneity test							Test of Association					Publication bias
Name	Explanation	Ethnicity	Q value	d.f.	I-squared	Tau-squared	*P* Value	Heterogeneity	Effect model	Pooled OR	95% CI	Z value	*P* value	Statistical significance	
Allele model	G vs. A	Caucasian	17.38	6	65.5%	0.0493	0.008	Yes	Random	0.985	[0.797, 1.217]	0.14	0.888	No	No
		Asian	14.90	7	53.0%	0.0564	0.037	Yes	Random	1.100	[0.871, 1.390]	0.80	0.421	No	
		Total	34.85	14	59.8%	0.0487	0.002	Yes	Random	1.037	[0.890, 1.210]	0.47	0.640	No	
Homozygote model	GG vs. AA	Caucasian	5.60	6	0.0%	NA	0.469	No	Random	1.119	[0.731, 1.714]	0.52	0.605	No	No
		Asian	10.22	7	31.5%	NA	0.176	No	Random	1.146	[0.743, 1.767]	0.62	0.538	No	
		Total	15.81	14	11.4%	NA	0.325	No	Random	1.118	[0.830, 1.506]	0.74	0.462	No	
Heterozygote model	AG vs. AA	Caucasian	16.71	6	64.1%	0.0575	0.010	Yes	Random	0.983	[0.780, 1.237]	0.15	0.882	No	No
		Asian	15.58	7	55.1%	0.1296	0.029	Yes	Random	1.433	[1.015, 2.023]	2.04	0.041	No	
		Total	42.72	14	67.2%	0.1017	0.000	Yes	Random	1.155	[0.935, 1.427]	1.34	0.181	No	
Dominant model	AG + GG vs. AA	Caucasian	41.43	8	80.7%	0.1518	0.000	Yes	Random	1.185	[0.882, 1.593]	1.13	0.259	No	No
		Asian	16.65	7	58.0%	0.1310	0.020	Yes	Random	1.379	[0.983, 1.934]	1.86	0.063	No	
		Total	63.64	16	74.9%	0.1467	0.000	Yes	Random	1.261	[1.008, 1.578]	2.03	0.042	No	
Recessive model	GG vs. AA + AG	Caucasian	5.24	6	0.0%	NA	0.513	No	Random	1.142	[0.746, 1.747]	0.61	0.542	No	No
		Asian	6.21	7	0.0%	NA	0.516	No	Random	0.919	[0.673, 1.255]	0.53	0.595	No	
		Total	12.06	14	0.0%	NA	0.602	No	Random	0.991	[0.771, 1.275]	0.07	0.946	No

The ethnicities are an Asian group and a Caucasian group. The corresponding results are shown in Table 6 and Figs. 3, 4, 5, 6, 7. For both the 2 subgroups, the OPRM1-A118G polymorphism had no association with alcohol-dependence in all these 5 genetic-models.

### Sensitivity analysis and publication bias

The ORs were not influenced by removing any single article (Fig. 2d–f). We had searched all possible studies both in Chinese databases and English databases to reduce the publication bias. Contour-enhanced funnel plots demonstrated that the studies only had missing areas for high statistical significance instead of low significance areas, thus very little or none publication bias was detected (Fig. 2g–i).

## Discussion

Alcohol dependence is estimated to exhibit heritability of more than 50% [5, 6], indicating genetic factors might play pivotal roles alcohol-dependence. Genome-wide or phenome-wide associations researches of alcohol-dependence was presented in Table 1. In view of the significances of μ-opioid receptor systems in physiologic mechanisms of reward centers, it is safe to say that OPRM1-polymorphisms had an influence on alcohol-dependence risks. [32, 33] Therefore, we focused our study on OPRM1 A118G, which is a functional allelic-variant with deleterious effects on protein and mRNA expressions. [34]

Close associations are suspected of the OPRM1 A118G polymorphism (A > G) with nicotine, alcohol, and opioid dependence. [13, 35, 36] Kapur et al. and Tan et al. discovered close associations between A118G-polymorphisms and heroin dependence. [37, 38] Modulation changes of kinase A are likely responsible for the close associations of the OPRM1 A118G polymorphism (A > G) with heroin dependence. [39] Recently, Frances et al. found that the OPRM1 A118G polymorphism (A > G) was associated with alcohol/tobacco-dependence in a Spanish population, and this association was related to several environmental and genetic factors. [18] However, the study from Rouvinen-Lagerstrom et al. suggested that the effect of A118G-polymorphism on the development of alcohol dependence was not statistically significant (*P* > 0.05). [40] In a study by Franke et al., data from ethnically homogenous samples detected no actual difference of the OPRM1 A118G polymorphism between alcohol dependent subjects and controls. [19]

We combed PubMed, Embase, Web of knowledge and CNKI databases in search of associations of alcohol dependence with the OPRM1 A118G polymorphism to cover the most information sourced from both Chinese and English studies. In our meta-analysis, significant associations between alcohol-dependence risks and A118G-polymorphisms were only found in the dominant model (OR 1.261, 95% CI 1.008, 1.578; *p* = 0.042). Association was non-significant in four other models. For subgroup analyses of Caucasian or Asian group each considered separately, the OPRM1 A118G polymorphism did not have association with alcohol dependence in all five genetic models.

In the contour-enhanced funnel plots, each circle represented a study. If studies appeared to be missing in areas of low statistical significance (the left part of the plot), the asymmetry is likely to be due to publication-biases. [41] In the present study, funnel plots indicated no publication bias.

There are potential limitations in our meta-analysis. The numbers of studies (nine and eight) as well as sample sizes for each ethnicity were limited. Type-II error could not be dismissed. [42] In addition, effects of gene-environment interactions and gene-gene interactions were not analyzed as not all eligible articles included these type of data. Within those studies with genomic interaction data, confounding factors were controlled and reported differently. Last, ORs adjusted by patient characteristics including genders, ages, living styles, medication-consumptions and other exposure-factors using meta-regression could be calculated with higher accuracy if related data were available in the majority of eligible studies.

## Conclusions

The opioid receptor mu 1 (OPRM1) A118G polymorphism (rs1799971) is not associated with alcohol dependence in Caucasian nor Asian populations.

## Abbreviations

95% CI: 95% confidence interval
CNKI: Chinese National Knowledge Infrastructure
HWE: Hardy-Weinberg equilibrium
MeSH: Medical Subject Heading
OPRM1: opioid receptor mu 1
OR: odds ratio
PCR-RFLP: polymerase chain reaction-restricted fragment length polymorphisms.
PRISMA: Preferred Reporting Items for Systematic Reviews and Meta-Analyses
